# Peters plus syndrome and absence of kidney: a case report

**DOI:** 10.1186/1757-1626-2-2

**Published:** 2009-01-01

**Authors:** Navneet Tuli, Suresh Kumar, Sunandan Sood

**Affiliations:** 1 Department of Ophthalmology, Government Medical College and Hospital, Chnadigarh, Indıa

## Abstract

A case of bilateral Peters anomaly with missing kidney on left was examined. The case of missing kidney in Peters anomaly has not been reported in literature to our knowledge, making this case a unique one.

## Background

Peters anomaly is a congenital condition characterized by iridocorneal contact glaucoma and corneal opacities usually in the absence of systemic anomalies. The condition usually results in loss of vision in the affected eye.

## Case report

**Figure 1:**
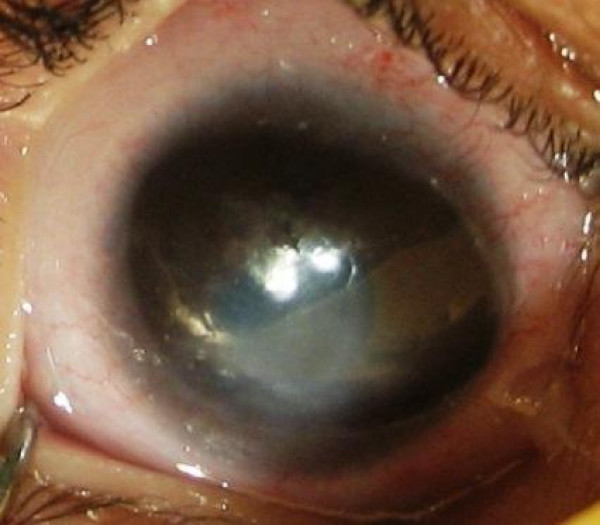
**Photograph of right eye**.

**Figure 2:**
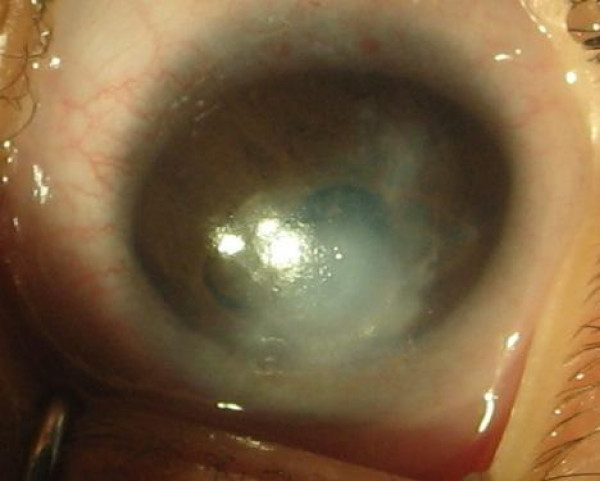
**Photograph of left eye**.

An 8 month old female infant presented with white discoloration of both eyes since birth. She had bilateral corneal opacity and nystagmus. Examination under anesthesia showed her corneal diameters to be large for her age (Horizontal diameter were 11.0 mm and 10.5 mm and vertical diameter were 10.5 mm and 10.0 mm of right and left eye). Schiotz Tonometry showed IOP of 29.7 mm of Hg in right eye (fig 1) and 25.1 mm of Hg in left eye (fig 2). The corneal opacity in right eye was central measuring 4 × 4 mm and in left eye whole of the cornea was involved leaving superior 3 mm of cornea clear. Bilateral multiple temporal and nasal iridocorneal adhesions could be appreciated. Lens was normal and B scan showed normal posterior segment. Systemic examination of respiratory, cardiovascular and central nervous system was normal but ultrasound abdomen showed absence of kidney on left side which was confirmed on CT abdomen. There was no history of consanguity of marriage in parents.

**Figure 3:**
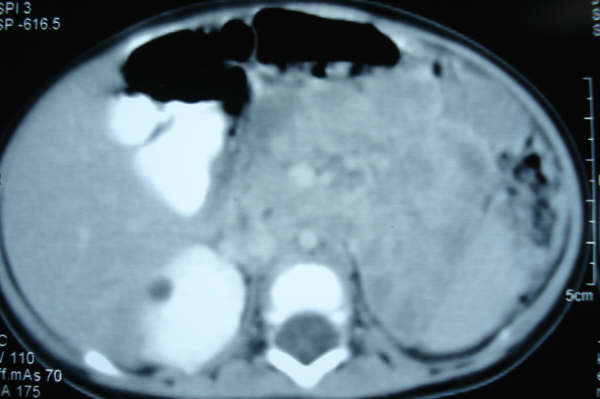
**CT Scan showing absence of left kidney**.

The absence of kidney represents a new association with Peters anomaly (Fig 3).

## Discussion

Peters anomaly can be inherited in autosomal dominant or autosomal recessive mode as well as occurring sporadically (with no other associated family members)[1]. It is associated with chromosome 4 abnormalities.[2] Systemic abnormalities include congenital heart defects, hydrocephalus, and renal dysgenesis.[3] Midline body structures seem to be selectively involved. Endocrinal anomalies result from association of Pituitary dysfunction.[4] Patients can have cleft lip and palate, genitourinary abnormalities, pilonidal cyst,[5] spina bifida, sacral hypoplasia, anal atresia or vesicocolonic fistula. The prevalence of Peters plus syndrome is unknown. Fewer than 70 affected individuals have been reported in the literature; they come from varied ethnicities.

Genitourinary abnormalities are reported in about 10–19% of patients suffering from Peter's anomaly. Genitourinary anomalies include hydronephrosis, renal and ureteral duplication, renal hypoplasia and oligomeganephroma, multicystic dysplastic kidney and glomerulocystic kidneys. [6] It has been determined that embryogenetic stages of eye and kidney development occur simultaneously. [7] From 7^th ^to 10^th ^week the development of ocular architecture progresses in parallel with the differentiation of kidney tubules. [8] It is conceivable that unknown factor leading to development of renal tubular cyst in utero might simultaneously affect eye and kidney. A variety of human congenital syndromes affected both organs have been described. Recently, analysis of spontaneous and transgenic mouse mutant has revealed that vertebrate pax genes are key regulators during organogenesis of kidney of eye. [9]

B3GALTL is the only gene known to be associated with Peters plus syndrome. Most affected individuals tested to date are homozygous for a hot spot splice mutation in intron 8 (c.660+1G > A). The parents of an affected child are obligate heterozygotes and thus carry one mutant allele. Heterozygotes (carriers) are asymptomatic. At conception, each sib of an affected individual has a 25% chance of being affected, a 50% chance of being an asymptomatic carrier, and a 25% chance of being unaffected and not a carrier. There is an increased chance for miscarriages and second- and third-trimester fetal loss of homozygously affected fetuses. Once an at-risk sib is known to be unaffected, the risk of his/her being a carrier is 2/3. The offspring of an individual with Peters plus syndrome are obligate heterozygotes (carriers) for a disease-causing mutation. Each sib of the proband's parents is at a 50% risk of being a carrier. Carrier testing for at risk family members is available on a clinical basis once the mutations have been identified in the family [6]. In most cases no prenatal diagnosis possible. However, in the minority where a gene defect can be identified, the prenatal diagnosis may be a possibile. [1]

Our case demonstrated absence of kidney on one side which we believe is unreported association. Patients with Peters anomaly should be screened for systemic malformations as therapeutic intervention for such problems is life saving.

## Consent

The written informed consent of the kin of the patient was taken.

## Competing interests

The authors declare that they have no competing interests.

## Authors' contributions

NT Case first seen in OPD and routine examination was done. Various differential diagnosis kept in mind and investigation ordered after consulting SK (Unit Incharge) and SS (Head of Department)

All the authors reviewed this case investigation and clinically till the final diagnosis was made.
